# Could *Pontimonas* Harbour Halophilic Members Able to Withstand Very Broad Salinity Variations?

**DOI:** 10.3390/microorganisms10040790

**Published:** 2022-04-08

**Authors:** Susanna Gorrasi, Marcella Pasqualetti, Martina Braconcini, Barbara Muñoz-Palazon, Massimiliano Fenice

**Affiliations:** 1 Department of Ecological and Biological Sciences (DEB), University of Tuscia, Largo Università snc, 01100 Viterbo, Italy; mpasqual@unitus.it (M.P.); martina.braconcini@unitus.it (M.B.); bmp@ugr.es (B.M.-P.); fenice@unitus.it (M.F.); 2 Laboratory of Ecology of Marine Fungi, CoNISMa, Department of Ecological and Biological Sciences, University of Tuscia, Largo Università snc, 01100 Viterbo, Italy; 3 Institute of Water Research, University of Granada, 18071 Granada, Spain; 4 Laboratory of Applied Marine Microbiology, CoNISMa, Università degli Studi della Tuscia, Largo Università snc, 01100 Viterbo, Italy

**Keywords:** *Pontimonas*, hypersaline environments, marine salterns, Saline di Tarquinia, metabarcoding survey

## Abstract

*Pontimonas* is currently described as a genus including only one species of slightly halophilic marine bacteria. Although some works revealed its presence in some hypersaline environments, the information on its habitat preference is still scant. This work investigated *Pontimonas* presence in selected ponds of the Saline di Tarquinia marine saltern and in the seawater intake area. The two-year metabarcoding survey documented its constant presence along the ponds establishing the salinity gradient and in a distinct basin with permanent hypersaline conditions (BSB). *Pontimonas* was higher in the ponds than in the sea, whereas it had similar abundances in the sea and in the BSB. Its representative OTUs showed significant trends according to different parameters. Along the salinity gradient, OTU1 abundance increased with decreasing water temperatures and increasing rainfalls, and it showed a maximum in January; OTU2 increased with increasing BOD_5_ and it showed the highest abundances in the period August–October, and OTU 3194 increased at decreasing salinities. In BSB, a significant seasonal variation was shown by OTU 3194, which started increasing in spring to reach a maximum in summer. The results suggest that *Pontimonas* could easily settle in hypersaline habitats, having also broad euryhaline members and some possible extreme halophilic representatives.

## 1. Introduction

*Pontimonas* is a genus of *Actinobacteria* that currently includes exclusively *p. salivibrio* as the sole described and validated species (List of Prokaryotic Names with Standing in Nomenclature, https://lpsn.dsmz.de/genus/pontimonas, (accessed on 1 February 2022)) [1]. The type strain *P. salivibrio* CL-TW6^T^ was isolated in Korea from a solar saltern pond with salinity slightly higher than seawater [2] and it represents the sole *Pontimonas* strain to have been cultured and characterised. Its growth occurred at 15–35 °C, pH 7.0–9.0, and sea salt concentrations of 10–90‰ (30 °C, 8.0, and 30‰ being the optimal values of temperature, pH, and salinity, respectively) [2,3].

The researchers who described the species assumed that *P. salivibrio* CL-TW6^T^ was a photoheterotrophic streamlined and slightly halophilic marine bacterium [4]. They supposed that it was dragged into the saltpan from the coastal area. The strain presence in a saltern could be explained by its halotolerance to the mentioned salt concentrations; however, it did not show tolerance to the highest salinities typical of extreme halophiles [4].

No other work has mentioned *Pontimonas* strains isolated from marine salterns or other hypersaline environments, and as far as we know, only the metabarcoding study provided by Leoni et al. [5] revealed the presence of *Pontimonas* (although with quite low relative abundance, ~2%) within a marine saltern at intermediate salinities (131‰ and 145‰).

Nevertheless, some studies carried out by culture-independent methods revealed its presence in other different talassohaline and atalassohaline environments with up to 185‰ salinity [6,7,8,9,10]. Moreover, it was found both in laboratory and productive aquaculture systems [11,12,13].

Due to the scarce literature availability, the information is insufficient to have an overall comprehension of *Pontimonas* ecology, and, in particular, to understand the habitat preferences of its members. Therefore, tracing the presence of *Pontimonas* in marine salterns is of valuable biological and ecological interest to contribute to fill this gap. Actually, if this genus were constantly found in marine salterns or other hypersaline environments, in a broad range of salinity, and with high abundances, the overall composition of the bacterial communities in these environments and their key members should be re-discussed. In fact, other taxa are generally found as dominant (e.g., *Spiribacter* and *Salinibacter*) and recognised as characteristic bacteria of these sites.

The “Saline di Tarquinia” (ST) are marine solar salterns located at ca. 80 km NW of Rome in the North Tyrrhenian Sea area. The ST system covers an area of ca. 135 ha, and it consists of ca. 100 shallow (15–50 cm) connected ponds, which establish an increasing salinity gradient [14]. Since 1980, ST have become a Nature Reserve. This led to a dramatic reduction in the anthropic activities (and consequent changes in the management of the area) [15] and some alterations in site fashioning [16]. Nonetheless, the ST system is an extreme environment, where rapid and wide-ranging changes in environmental parameters (particularly salinity and water temperature) are recorded in most of the ponds.

ST also hosted a large pool (~1 ha, 2–3 m depth), employed as brine storage basin (BSB) during the saltern activity period. BSB has been disconnected from the pond system following the salt production dismission. Currently, it represents a unique distinct ecosystem characterised by permanent hypersaline conditions [17].

Apart from the early work of Alfinito et al. [18] dealing with the ST microalgal flora, a more systematic study on the microbial ST communities started in 2010. Few studies regarded the presence and characterisation of phytoplanktonic organisms [19,20,21]. The research activities focused also on the characterisation of the ST bacterial communities, carried out both by culture-dependent and culture-independent methods [14,15,16,17,22,23].

Among them, some investigations evidenced the presence of interesting microorganisms with peculiar growth profiles in relation to the salinity or temperature variations [16]. Other studies suggested the ST system as a possible reservoir of microorganisms of public concern [14,17] or evidenced the constant occurrence (with high abundances) of bacteria whose distribution and ecology are still little known [15]. Among them, *Pontimonas* appeared to be one of the most represented bacterial genera. However, its distribution was not analysed in detail, particularly studying the pattern distribution of its different OTUs also in relation to different environmental conditions. In addition, its presence in BSB was never investigated.

The current work specifically investigated the spatio-temporal occurrence of *Pontimonas* OTUs along the ST salinity gradient. *Pontimonas* presence was also studied in different hypersaline environments comparing the BSB, a quite stable hypersaline habitat, with various ST ponds characterised by very variable environmental conditions. The investigation, involving a two-year sampling, was carried out through a metabarcoding survey.

## 2. Materials and Methods

### 2.1. Sample Collection and Characterisation

**Figure 1:**
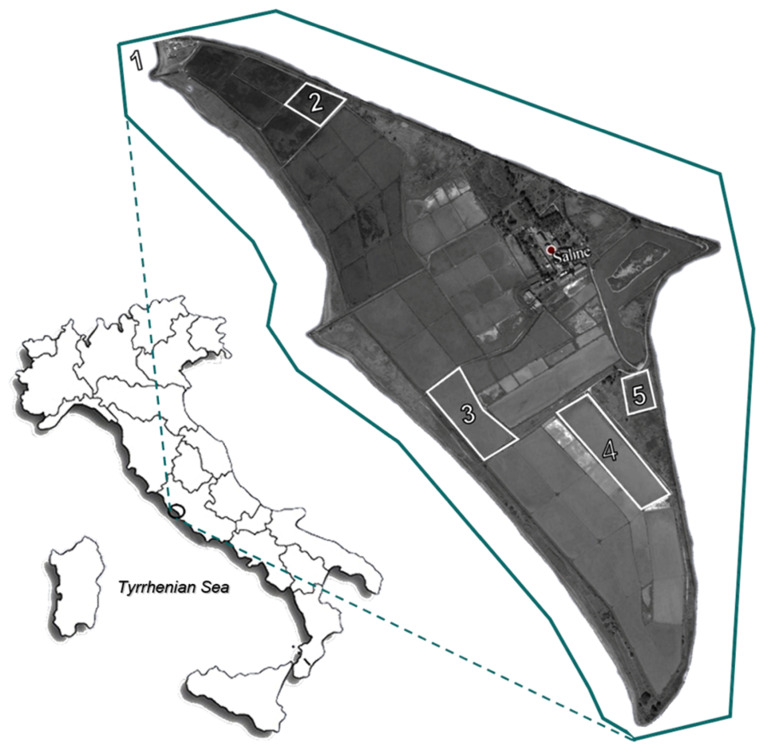
Map of the “Saline di Tarquinia” (Viterbo, Italy) salterns reporting the sampling sites. 1 = S, sea; 2 = P5, low-salinity concentrator pond; 3 = P24, intermediate-salinity concentrator pond; 4 = P37, crystallisation pond; and 5 = BSB. The map was acquired from Google Earth Pro (version 7.3.1) and graphically edited.

Monthly sampling was executed over 2 years (May 2012–April 2014) within the ST (North Tyrrhenian Sea, Italy; 42°12′07.8″ N 11°43′17.8″ E). The water samples were collected from the coastal area where the seawater is loaded into the ST system (referred to as “Sea”, S), representing the saltpan seawater inputs, from three different ponds and the brine storage basin (BSB). Three distinct non-consecutive ponds (P5, P24, and P37, representing a low-salinity concentrator pond, an intermediate-salinity concentrator pond, and a crystallisation pond, respectively) were selected (as a function of their annual range of salinity) to fully cover the ST gradient (Figure 1).

The sampling and the environmental parameter determination were performed as previously reported [15]. Three different sub-samples were collected at each sampling site and pooled to have a representative sample [24]. The samples were kept in sterile bottles at 4 °C to be transported to the nearby ST laboratory and processed within an hour. For each sample, 1 L of water was vacuum filtered on sterile filters (0.22 µm, Millipore, Burlington, MA, USA); then, to remove possible nutrients from the membranes, they were washed twice with saline sterile solutions (the NaCl concentrations corresponded roughly to the salinity recorded on the sites) [25]. The filters were kept frozen until DNA extraction. The other sample aliquots were intended for the BOD_5_ determination, which was carried out according to the standard methods [26].

The environmental parameters such as salinity, pH, conductivity, and water temperature were recorded during sampling using common handheld probes, whereas the daily rainfall data were taken from the database of a local government institution [27]. All environmental parameter data are reported in Table S1.

### 2.2. DNA Extraction, 16S rDNA Amplicon Library Preparation and Sequencing

Total DNA was extracted processing the filters through the ZR Fungal/Bacterial DNA MiniPrep kit (Zymo Research Corp., Irvine, CA, USA), according to the manufacturer’s instructions.

The multiplexed libraries of amplicons (V5-V6 hypervariable regions of the 16S rDNA) were prepared using a dual PCR amplification protocol. The strategy adopted for the amplicon library preparation, as well the primer set used and the protocols, have already been described in detail elsewhere [15]. The Illumina sequencing (MiSeq system, Illumina, San Diego, CA, USA; 2 × 250 bp paired-end protocol) was carried out at Nuova Genetica Italiana SRL (Monza-Brianza, Italy).

### 2.3. Sequence Processing and Data Analysis

Sequence processing and data analysis were performed as reported by Gorrasi et al. [17]. First, the raw reads obtained from the sequencing were demultiplexed according to the indices and the internal barcodes, and then processed applying the UPARSE pipeline [28]. Forward and reverse reads were merged (with perfect overlap) and quality filtered using the default parameters. The suspected chimeras and the singleton sequences (appearing only once in the entire dataset) were removed. OTUs were defined on the entire dataset clustering the sequences at 97% of similarity; for each cluster, a representative sequence was defined. The OTU abundances per sample were estimated by mapping the sequences to the OTU representative sequences (at 97% of similarity). The taxonomic assignation was performed using RDP classifier [29], applying a 50% confidence cut-off as suggested for short sequences [30].

### 2.4. Statistical Methods

The Redundancy Analysis (RDA) was run to individuate the environmental parameters affecting the *Pontimonas* community. For the RDA analysis, the OTU abundance data were square root transformed. The environmental parameters tested were: sampling site, salinity, pH, water temperature, BOD_5_, sampling month (entered as Fourier series transformed data, *sin*(Month) and *cos*(Month), to account for seasonality) [31], and rain (cumulative data related to the seven days preceding the sampling).

The Pearson matrix was calculated to inspect collinear variables (Pearson |r| > 0.6). For the stepwise selection of the explanatory variables, we ran the forward selection and corrected the significance according to the False Discovery Rate (FDR), to avoid Type I error inflation [32]. The variables collinear with the predictors or not significant according to the forward selection were removed from the RDA analysis. The RDA significance was evaluated by the Monte Carlo permutation test, using 9999 permutations.

The Generalised Linear Models (GLMs) were run to investigate the abundance variation of the most abundant OTUs according to the key environmental parameters, assuming a Poisson distribution with a log link function. The periodic regression based on Fourier series transformation of the sampling month data was used to investigate the OTU seasonal variation.

The Pearson matrix was calculated using the statistical software Systat 8.0 (Systat Software Inc., Point Richmond, Richmond, CA, USA); the PCA, RDA, and GLM analyses were performed using CANOCO v. 5.1 (Microcomputer Power, Ithaca, NY, USA).

To select the appropriate tests to assess the pairwise differences in genus/OTU abundance variation among the sampling sites, the data normal distribution and the homogeneity of variance assumptions were firstly tested (by Shapiro–Wilk and Levene tests, respectively). Since the assumptions were not met, pairwise comparison was performed using the non-parametric test Kruskal–Wallis One-Way Analysis of Variance followed by the Kolmogorov–Smirnov Two Sample Test. All these tests were run using Systat 8.0.

## 3. Results

### 3.1. Spatio-Temporal Variation in Pontimonas

In summary, a total of 3,248,348 bacterial sequences were obtained after quality control, and, among them, 569,318 were assigned to *Pontimonas*. The sequence clustering process returned 111 OTUs ascribed to this genus.

*Pontimonas* was constantly detected (over two years) along the ST salinity gradient (from the sea up to the crystallisation pond) and in the BSB (Figure S1). Except in June 2012, July 2013, and March 2014, when *Pontimonas* was higher in the sea (S) than in P37, its abundance was always lower in the sea than in the various ponds. Differently from the sea samples, in those collected from the ponds it always had relative abundance (Ra) > 1%, and it was detected with very high abundances mainly in P5 and P24. The ranges of the relative abundances (Ras) were 0.3–19.5%, 10.8–61.7%, 8.4–85.8%, and 1.2–65.9% in S, P5, P24, and P37, respectively.

In the sea, a general increase in its abundance was recorded in the second sampling year compared to the first year, with a peak in June 2013. In P5 (low-salinity concentrator pond), it showed abundance almost always quite high over the two years; its highest abundances (>50%) were in February, June, and September 2013. In P24 (intermediate-salinity concentrator pond) it showed a high abundance increase in the second year of the sampling campaign; it was recorded with the highest Ras (>70%) in September–October 2013 and in January 2014. In P37 (crystalliser pond), it showed a general abundance increasing over the two years, and it raised the highest Ras (>50%) in November 2013, January, and April 2014.

**Figure 2:**
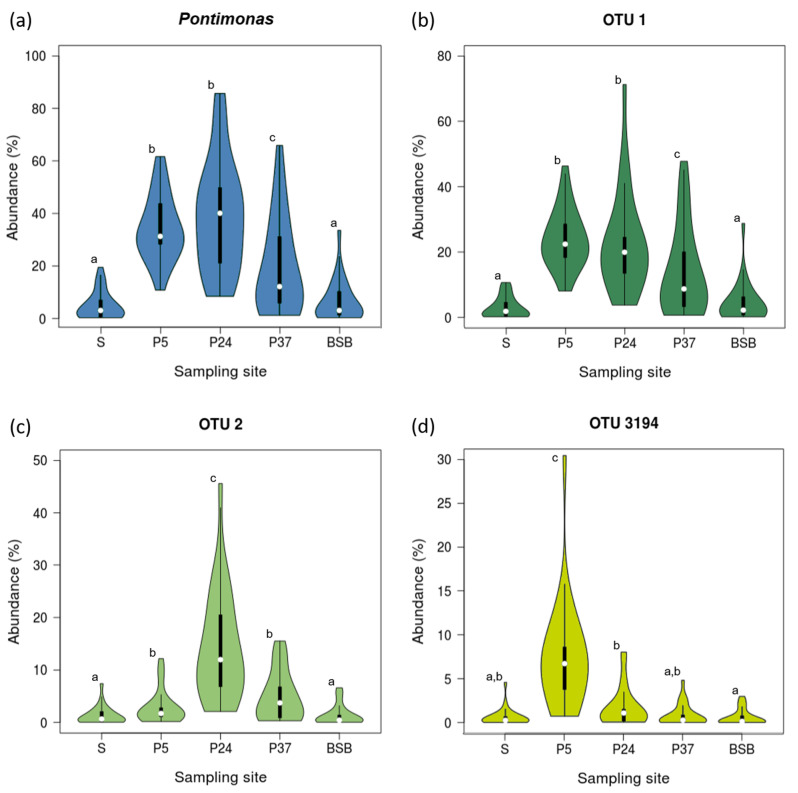
Violin plots showing the abundance variation of *Pontimonas* (**a**) and its most abundant OTUs (**b**–**d**) among the ST sampling sites in the period May 2012–April 2014. The same superscript letters indicate no significant differences between the mean values of different groups (Kruskal–Wallis followed by Kolmogorov–Smirnov post hoc test).

In most of the sampling months (14 out of 24), *Pontimonas* abundance increased along the salinity gradient up to the intermediate-salinity concentrator pond, to decrease thereafter in the crystallisation pond. An increase in its abundance along the whole gradient (up to the crystallisation pond) was evidenced only in November 2013 and April 2014. Instead, a *Pontimonas* decrease along the salinity gradient was observed in the periods August–November 2012 and February–April 2013, and in June 2013. The analysis of global (cumulative) data showed an overview of *Pontimonas* occurrence over the biennial survey: the taxon showed the lowest abundance in S, to markedly increase in P5 and P24 (no statistically significant differences were recorded between these two sites; *p* = 0.378), and to decrease in P37 (Figure 2a).

In BSB (the hypersaline basin not connected to the ST pond system), *Pontimonas* was detected with Ras ranging from 0.3 to 33.6%, and higher abundances were recorded in the second sampling year than in the first year. It occurred with the highest Ras (≥10%) in the period May–July 2013, in November 2013, and April 2014.

Furthermore, its abundance in BSB over the two years (cumulative data) was similar (*p* = 0.604) to that recorded in the sea (Figure 2a).

**Figure 3:**
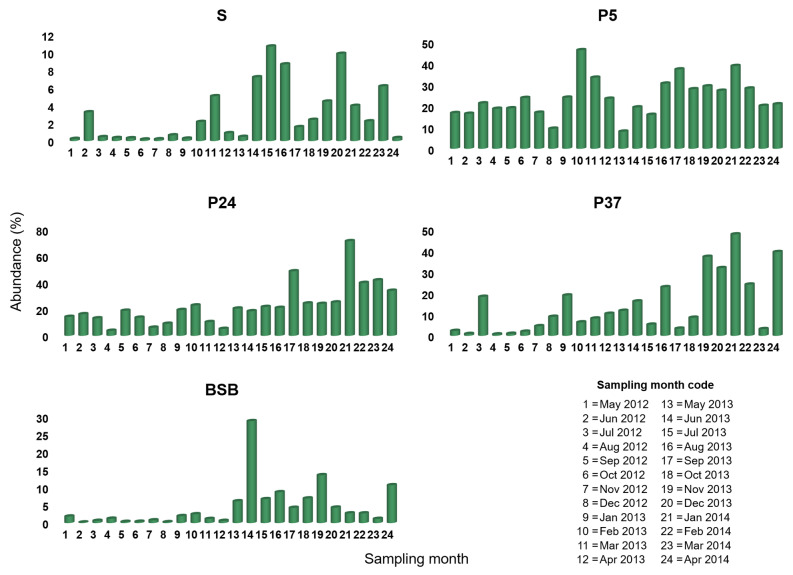
Occurrence of OTU 1 among the ST sampling sites in the period May 2012–April 2014.

**Figure 4:**
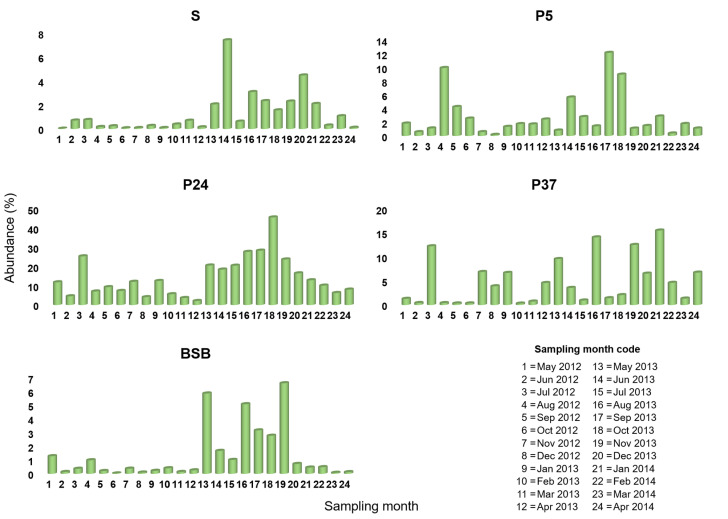
Occurrence of OTU 2 among the ST sampling sites in the period May 2012–April 2014.

**Figure 5:**
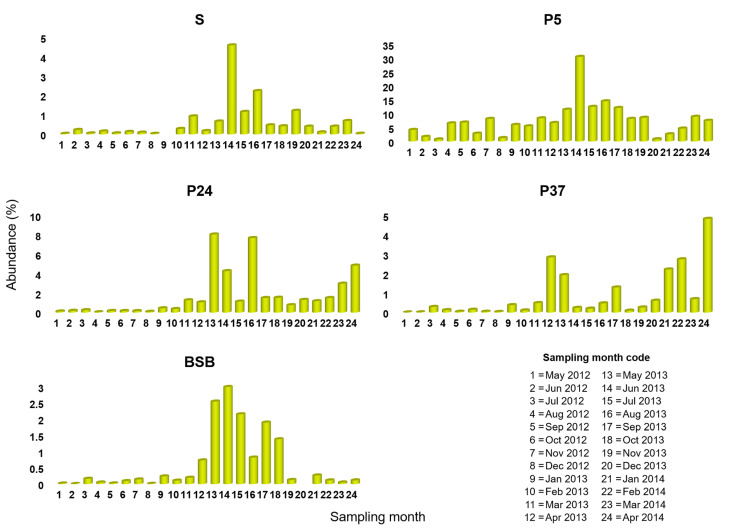
Occurrence of OTU 3194 among the ST sampling sites in the period May 2012–April 2014.

Among the 111 *Pontimonas* OTUs, OTU 1, OTU 2, and OTU 3194 were the most abundant, including the 64.5%, 24.3%, and 10.8% of the *Pontimonas* sequences mapped in the whole dataset, respectively. Monthly data revealed a different occurrence pattern of these OTUs (Figure 3, Figure 4 and Figure 5) along the salinity gradient and in BSB. In particular, it was evident that OTU1 and OTU 3194 together largely contributed to the *Pontimonas* temporal pattern observed in P5 (low-salinity concentrator pond), whereas OTU 1 and OTU 2 to the genus temporal variability observed in P24/P37 (intermediate-salinity concentrator/crystallisation pond). In BSB, OTU1 was the principal OTU determining the *Pontimonas* temporal pattern (Figure 3).

Along the salinity gradient, OTU 1 was detected with Ras in between 0.2–10.7%, 8.0–43.6%, 0.7–47.8%, and 0.2–28.8% in S, P5, P24, and P37, respectively (Figure 3). OTU 1 abundances were higher in the ST ponds than in the sea, and in general, its pattern along the salinity gradient reflected that of the whole genus (Figure 3 and Figure S1). In P5, it showed the highest Ras (>25%) in the periods February–March 2013 and August 2013–February 2014. In P24, it was detected with Ras constantly greater than 20% in the second sampling year, showing two peaks in September 2013 (48.6%) and January 2014 (71.3%). In P37, except for some months, it showed a general temporal abundance increasing; it occurred with the highest abundances (>30%) in the period November 2013–January 2014 and in April 2014. In BSB it showed higher abundances. In BSB, it was mainly recorded (in 17 out 24 months) with Ras < 5%, and it showed a peak (28.8%) in January 2013.

Observing OTU 2 and OTU 3194 patterns along the salinity gradient, their occurrence with higher abundances was evident in the intermediate-salinity and low-salinity concentrator pond, respectively (Figure 4 and Figure 5).

OTU 2 and OTU 3194 showed a notable peak in October 2013 in P24 and in June 2013 in P5, respectively. In addition, in BSB they were mostly recorded with Ras ≤ 1%; over the two years, OTU 2 had Ras in the range 1.0–6.6% in nine months and OTU 3194 had Ras in the range 1.4–3.0% in five months.

Overall, a global analysis of the biennial data showed that OTU 1 and OTU 2 abundances were significantly higher in the ponds than in the sea (*p* < 0.05) (Figure 2b,c), whereas OTU 3194 showed similar abundances in the sea, the intermediate-salinity concentrator (P24), and crystallisation ponds (P37) (Figure 2d). OTU 1 showed an abundance decrease along the salinity gradient (from P5 to P37) (Figure 2b); OTU 2 and OTU 3194 had abundances significantly higher (*p* < 0.05) in P24 and P5, respectively (Figure 2c,d). Moreover, all the three OTUs had similar abundances (*p* > 0.05) in BSB and S.

### 3.2. Environmental Parameters Affecting Pontimonas Occurrence

The RDA was run to investigate the parameters affecting the *Pontimonas* spatio-temporal pattern along the salinity gradient and in BSB, and to understand the relationships between the most abundant OTUs occurrence and these parameters. Since BSB is a separated ecosystem, without water exchanges with the pond system, the RDA was performed analysing the data collected along the salinity gradient and those collected in BSB separately. 

As for the analysis of the data collected along the salinity gradient, among the environmental factors taken into account, water temperature, pH, and conductivity were excluded from the RDA since they were collinear with other parameters (Pearson |r| > 0.6). The tested parameters were: sampling site, salinity, BOD_5_, sampling month (entered as *sin*(Month) and *cos*(Month) to account for seasonality), and rain. Along the salinity gradient, *Pontimonas* community changed significantly in relation to the sampling site, salinity, BOD_5_, and *sin*(Month) (Table 1).

Among the significant parameters, the sampling site principally affected the spatio-temporal distribution of *Pontimonas* along the salinity gradient; it explained the highest percentage of total variance (60.7%, sum of the variance percentages explained by each sampling site; Table 1).

**Figure 6:**
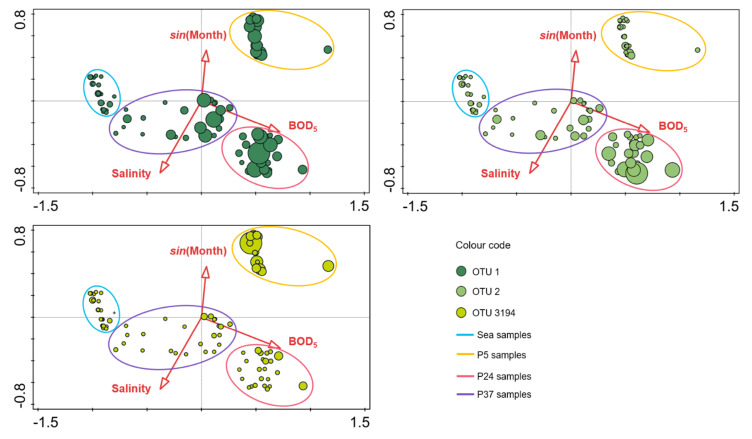
RDA analysis based on OTU composition of the *Pontimonas* communities found along the ST salinity gradient. Each triplot evidences the occurrence of one of the three most abundant OTUs. Circle size is proportional to the OTU relative abundance in the samples; OTU absence in a sample is indicated by “+”.

The RDA triplot (Figure 6) showed that the samples formed distinct clusters based on the sampling sites. In addition, the *Pontimonas* communities in S, P5, and P24 samples were related to low BOD_5_, low salinity, and high BOD_5_, respectively. Three RDA triplots were presented to better appreciate the different parameter contribution to the occurrence of each most abundant OTU. OTU 1 and OTU 2 occurrence in P24 was mainly affected by nutrient (organic substance) availability: their highest abundances were in P24 samples characterised by the highest BOD_5_ values. Moreover, OTU 1 showed quite high abundances in P5 samples characterised by low salinities and in some P37 samples collected characterised by low values of salinity and BOD_5_. OTU 3194 occurrence seemed to be mainly affected by the salinity, being more abundant in P5 samples characterised by low salinities.

Although some parameters were not significant for the RDA model, based on the contribution of all *Pontimonas* OTUs, the GLMs were performed analysing the trend of the most abundant OTUs versus all parameters, to understand whether the OTU abundance variation (along the salinity gradient) in relation to them could still be significant.

**Figure 7:**
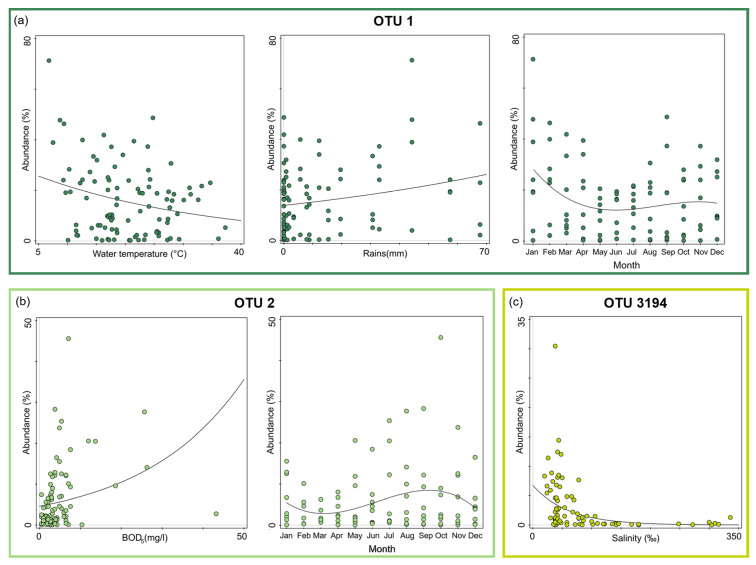
GLM plots showing the significant trends of the most abundant OTUs in relation to the environmental parameters along the ST salinity gradient. OTU 1 abundance variation according to the water temperature, the rain, and the sampling month (**a**); OTU 2 abundance variation according to the BOD_5_ and the sampling month (**b**); OTU 3194 abundance variation according to the salinity (**c**).

The GLMs analyses revealed that OTU 1 showed a significant abundance variation in relation to the water temperature (F = 6.9, *p* = 0.010) and the rain (F = 4.8, *p* = 0.030): it decreased at increasing water temperature and increased with the rainfall increase (Figure 7a). In addition, OTU 1 was characterised by seasonal variation (*cos*(Month): F = 5.6, *p* = 0.020): it had a maximum in January to decrease and reach a minimum in late spring, and slightly increasing again in autumn (Figure 7a).

OTU 2 showed a significant abundance variation in relation to the organic substance availability (BOD_5_: F = 7.1, *p* = 0.009) and the seasonality (*sin*(Month): F = 4.5, *p* = 0.036). Its abundance increased with increasing BOD_5_; moreover, during the periods February–April and August–October it showed the lowest and the highest abundances, respectively (Figure 7b).

OTU 3194 revealed a significant abundance variation in relation to the salinity (F = 13.7, *p* = 0.0004), decreasing at increasing salinities (Figure 7c).

As for the analysis of the data collected in BSB, water temperature, pH, and conductivity were excluded from the RDA since they were collinear with other parameters (Pearson |r| > 0.6). The tested parameters were: sampling site, salinity, BOD_5_, sampling month (entered as *sin*(Month) and *cos*(Month) to account for seasonality), and rain.

**Figure 8:**
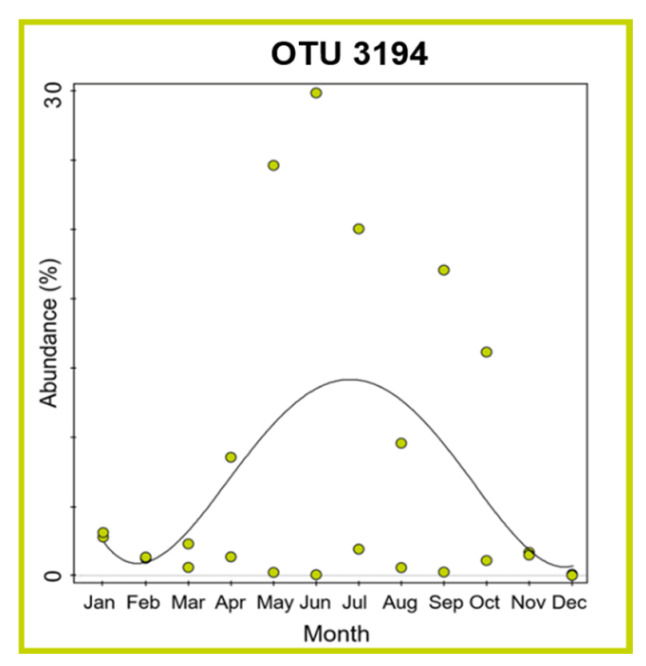
GLM plot showing the significant abundance variation of OTU 3194 in relation to the sampling month in BSB.

The RDA analysis evidenced that none of the considered parameters significantly affected the temporal dynamic of *Pontimonas* communities in BSB. However, the GLM analyses revealed a significant trend in relation to the seasonality for OTU 3194 (*cos*(Month): F = 9.2, *p* = 0.006): it started increasing in spring to reach a maximum in summer, and to decrease again in autumn (Figure 8).

## 4. Discussion

*Pontimonas* is considered a genus of marine bacteria that, although discovered in 2013, appeared to be quite neglected by the scientific community. Very little is known about its presence in marine and/or hypersaline environments, and its biology and ecology are far from being sufficiently understood. Therefore, tracing *Pontimonas* presence in this extreme environment is of valuable scientific interest. Therefore, the current study aimed to investigate its presence in ST marine salterns. Its spatio-temporal occurrence over two years along the ST salinity gradient and in BSB was investigated through a metabarcoding survey.

Although with marked differences related to both the sampling site and month, the two-year metabarcoding survey carried out in this work evidenced a constant presence of *Pontimonas* along the salinity gradient and in BSB. The RDA analysis showed that the spatio-temporal distribution of *Pontimonas* along the salinity gradient was mainly affected by the sampling site, then by salinity, BOD_5_, and *sin*(Month) (Table 1); whereas none of the parameters taken into account significantly affected the *Pontimonas* community dynamic in BSB. On the whole, it is interesting to note that *Pontimonas* showed higher abundances in the saltern ponds (the highest abundances being recorded in the low- and intermediate-salinity concentrator ponds), rather than in the sea. It was recorded with notable abundances (72–79%) in P24 samples characterised by 92‰ and 106‰ salinity (Figure S1 and Table S1). As mentioned, according to the studies of Jang et al. [2], *Pontimonas* is a marine bacterium drawn into a saltern system from the nearby coastal environment. Moreover, the strain described by these authors is a slight halophilic bacterium. Our outcomes indicated that within ST this genus found favourable conditions supporting its settling, also at intermediate or even high salinities. Moreover, although with abundances overall similar to those recorded in the sea (Figure 2), it was always found in the hypersaline basin. Here, it was detected with quite high Ra (33.6%) at 208‰ of salinity (June 2013) and showed Ra of 9.4% in September 2013, when the salinity maximum (~360‰) was achieved (Figure S1 and Table S1). Furthermore, also in the crystallisation pond (P37), it was found at very high salinity (300–310‰) with quite high Ra (30–38%) (Figure S1 and Table S1). All this suggested the existence also of moderate halophilic representatives of the genus. The presence of *Pontimonas* in some hypersaline environments at intermediate salinities (between 110 and 145‰) [5,7] might support this hypothesis.

Considering the most abundant *Pontimonas* OTUs, different distribution patterns were evidenced along the ST salinity gradient and in BSB. In addition, their abundances varied significantly according to different environmental parameters and the seasonality. Along the salinity gradient, OTU 1 showed a maximum in January and its abundance increased with decreasing water temperature and increasing rainfall. OTU 2 showed the highest abundances in the period August–October and increased with increasing BOD_5_. Finally, OTU 3194 increased at decreasing salinities (Figure 7); no significant variation according to seasonality was recorded. In BSB, the only significant trend was shown by OTU 3194 in relation to the seasonality: its abundance increased in spring to reach a maximum in summer (Figure 8). The RDA evidenced that the relative distribution of the three OTUs in the ponds was mainly related to different variables (Figure 6). OTU1 showed the highest abundance in P5, P24, and P37 samples characterised by low salinities, high BOD_5_ values, and low BOD_5_ values, respectively. OTU 2 showed the highest abundance in P24 samples, having the highest BOD_5_ values and OTU 3194 in the P5 samples, characterised by low salinities. Although the salinity was among the parameters structuring the *Pontimonas* communities along the salinity gradient, a significant abundance variation in relation to this key parameter was evidenced only for OTU 3194 (Figure 7c). However, in general, all three OTUs showed higher abundances in the ponds than in the sea (Figure 2); in addition, they were found at all recorded salinities (20–360‰) both along the salinity gradient and in BSB (Figure 3, Figure 4 and Figure 5 and Table S1). Moreover, OTU 1 and OTU 2 were abundant also at high salinities. For instance, OTU 1 showed abundances of 22.9% and 18.4% in Aug13-P37 and Jul12-P37 samples, characterised by salinities of 300‰ and 310‰, respectively. Therefore, the presence of the most representative OTUs at all salinities (up to saturation), with a broad euryhalinism, suggested their ability to growth in hypersaline environments, indicating that *Pontimonas* members can be both slight and moderate halophilic bacteria, able to also tolerate high salinities.

## 5. Conclusions

Our work represents the first attempt to investigate its presence in a hypersaline environment, also in comparison with the neighbouring sea. This extensive survey evidenced that various OTUs representative of this genus are a constant presence in the studied ecosystems. The results suggest that species of this genus could be well adapted not only to sea environments, but also to hypersaline habitats, with possible presence of broad euryhalines strains and even some extreme halophilic ones.

## Figures and Tables

**Table 1:** Descriptive statistics of RDA analysis on samples collected along the salinity gradient.

Variable	Explains %	Contribution %	F	P	P (adj) ^1^
Sampling Site.S	29.4	50.0	39.2	0.00094	0.00189
Sampling Site.P5	15.7	26.7	26.7	0.00094	0.00113
Sampling Site.P37	7.8	13.2	15.2	0.00094	0.00113
Sampling Site.P24	7.8	13.2	15.2	0.00094	0.00113
Salinity	2.8	4.8	5.8	0.00754	0.00905
BOD_5_	1.6	2.7	3.3	0.03110	0.03110
*sin*(Month)	1.5	2.6	3.3	0.03770	0.03770

^1^ corrected according to the FDR.

## Data Availability

The sequence data are available at the European Nucleotide Archive (ENA), study accession number PRJEB38856 (http://www.ebi.ac.uk/ena/data/view/PRJEB38856, accessed on 1 February 2022).

## Supplementary Materials

The following supporting information can be downloaded at: https://www.mdpi.com/2076-2607/10/4/790/s1, Figure S1. Occurrence of *Pontimonas* among the ST sampling sites in the period May 2012–April 2014, Table S1: Environmental parameters recorded for the ST samples from May 2012 to April 2014.

## References

[1] LPSN-List of Prokaryotic Names with Standing in Nomenclature, Family Enterobacteriaceae. https://lpsn.dsmz.de/genus/pontimonas.

[2] Jang G.I., Cho Y., Cho B.C. (2013). *Pontimonas salivibrio* gen. nov., sp. nov., a new member of the family *Microbacteriaceae* isolated from a seawater reservoir of a solar saltern. Int. J. Syst. Evol. Microbiol..

[3] Cho B.C., Jang G.I., Hwang C.Y., Whitman W.E.B. (2015). Pontimonas. Bergey’s Manual of Systematics of Archaea and Bacteria.

[4] Cho B.C., Hardies S.C., Jang G.I., Hwang C.Y. (2018). Complete genome of streamlined marine actinobacterium *Pontimonas salivibrio* strain CL-TW6T adapted to coastal planktonic lifestyle. BMC Genom..

[5] Leoni C., Volpicella M., Fosso B., Manzari C., Piancone E., Dileo M.C.G., Arcadi E., Yakimov M., Pesole G., Ceci L.R. (2020). A differential metabarcoding approach to describe taxonomy profiles of bacteria and archaea in the saltern of Margherita di Savoia (Italy). Microorganisms.

[6] Al-Mailem D.M., Kansour M.K., Radwan S.S. (2017). Capabilities and limitations of DGGE for the analysis of hydrocarbonoclastic prokaryotic communities directly in environmental samples. Microbiol. Open.

[7] Selivanova E.A., Poshvina D.V., Khlopko Y.A., Gogoleva N.E., Plotnikov A.O. (2018). Diversity of prokaryotes in planktonic communities of Saline Sol-Iletsk lakes (Orenburg Oblast, Russia). Microbiology.

[8] Shurigin V., Hakobyan A., Panosyan H., Egamberdieva D., Davranov K., Birkeland N.K. (2019). A glimpse of the prokaryotic diversity of the Large Aral Sea reveals novel extremophilic bacterial and archaeal groups. Microbiol. Open.

[9] Skorupa D.J., Akyel A., Fields M.W., Gerlach R. (2019). Facultative and anaerobic consortia of haloalkaliphilic ureolytic micro-organisms capable of precipitating calcium carbonate. J. Appl. Microbiol..

[10] Çelik P.A., Mutlu M.B., Korkmaz F., Yaman B.N., Gedikli S., Çabuk D.D.A. (2021). Boron mine ponds: Metagenomic insight to bacterial diversity. Biol. Divers. Conserv..

[11] Quiroz M., Triadó-Margarit X., Casamayor E.O., Gajardo G. (2015). Comparison of *Artemia*–bacteria associations in brines, laboratory cultures and the gut environment: A study based on Chilean hypersaline environments. Extremophiles.

[12] Xue M., Liang H., He Y., Wen C. (2016). Characterization and in-vivo evaluation of potential probiotics of the bacterial flora within the water column of a healthy shrimp larviculture system. Chin. J. Oceanol. Limnol..

[13] Hozumi A., Hong P.Y., Kaartvedt S., Røstad A., Jones B.H. (2018). Water quality, seasonality, and trajectory of an aquaculture-wastewater plume in the Red Sea. Aquac. Environ. Interac..

[14] Gorrasi S., Pasqualetti M., Franzetti A., Gonzalez-Martinez A., Gonzalez-Lopez J., Muñoz-Palazon B., Fenice M. (2021). Persistence of *Enterobacteriaceae* drawn into a marine saltern (Saline di Tarquinia, Italy) from the adjacent coastal zone. Water.

[15] Gorrasi S., Franzetti A., Ambrosini R., Pittino F., Pasqualetti M., Fenice M. (2021). Spatio-temporal variation of the bacterial communities along a salinity gradient within a thalassohaline environment (Saline di Tarquinia salterns, Italy). Molecules.

[16] Barghini P., Pasqualetti M., Gorrasi S., Fenice M. (2018). Bacteria from the “Saline di Tarquinia” marine salterns reveal very atypical growth profiles with regards to salinity and temperature. Mediterr. Mar. Sci..

[17] Gorrasi S., Pasqualetti M., Franzetti A., Pittino F., Fenice M. (2020). *Vibrio* communities along a salinity gradient within a marine saltern hypersaline environment (Saline di Tarquinia, Italy). Environ. Microbiol..

[18] Alfinito S., Iberite M., Fumanti B. (1990). The algal microflora of the salt works of Tarquinia (Italy). Hydrobiology.

[19] Pasqualetti M., Bernini R., Carletti L., Crisante F., Tempesta S. (2010). Salinity and nitrate concentration on the growth and carotenoids accumulation in a strain of *Dunaliella salina* (Chlorophyta) cultivated under laboratory conditions. Transit. Water. Bull..

[20] Tempesta S., Paoletti M., Pasqualetti M. (2010). Morphological and molecular identification of a strain of the unicellular green alga *Dunaliella* sp. isolated from Tarquinia Salterns. Transit. Water. Bull..

[21] Barghini P., Giovannini V., Fenice M., Gorrasi S., Pasqualetti M. (2018). High lutein production by a halo-tolerant strain of *Dunaliella* sp. (chlorophyceae) isolated from solar salterns in central Italy. J. Environ. Prot. Ecol..

[22] Barghini P., Silvi S., Aquilanti A., Marcelli M., Fenice M. (2014). Bacteria from marine salterns as a model of microorganisms adapted to high environmental variations. J. Environ. Prot. Ecol..

[23] Barghini P., Pasqualetti M., Gorrasi S., Fenice M. (2018). Study of bacterial diversity of a saltern crystallisation pond (“Saline di Tarquinia”, Italy) and its correlation with salinity variations. J. Environ. Prot. Ecol..

[24] Gorrasi S., Pesciaroli C., Barghini P., Pasqualetti M., Fenice M. (2019). Structure and diversity of the bacterial community of an Arctic estuarine system (Kandalaksha Bay) subject to intense tidal currents. J. Mar. Syst..

[25] Gorrasi S., Pesciaroli C., Barghini P., Pasqualetti M., Giovannini V., Fenice M. (2019). Metagenetic profiling of the bacterial communities of an intertidal pool in Kandalaksha Bay (White Sea, Russia). J. Environ. Prot. Ecol..

[26] Rice E.W., Baird R.B., Eaton A.D., Clesceri L.S. (2012). Standard Methods for the Examination of Water and Wastewater.

[27] Centro Funzionale Regionale, Regione Lazio, Annali. http://www.idrografico.regione.lazio.it/annali/index.htm.

[28] Edgar R.C. (2013). UPARSE: Highly accurate OTU sequences from microbial amplicon reads. Nat. Methods.

[29] Wang Q., Garrity G.M., Tiedje J.M., Cole J.R. (2007). Naïve Bayesian classifier for rapid assignment of rRNA sequences into the new bacterial taxonomy. Appl. Environ. Microbiol..

[30] Claesson M.J., O’Sullivan O., Wang Q., Nikkilä J., Marchesi J.R., Smidt H., de Vos W.M., Ross R.P., O’Toole P.W. (2009). Comparative analysis of pyrosequencing and a phylogenetic microarray for exploring microbial community structures in the human distal intestine. PLoS ONE.

[31] Legendre P., Legendre L. (2012). Numerical Ecology.

[32] Verhoeven K.J.F., Simonsen K.L., McIntyre L. (2005). Implementing false discovery rate control: Increasing your power. Oikos.

