# Supplementary figures and images for: Could Pontimonas Harbour Halophilic Members Able to Withstand Very Broad Salinity Variations?
Source: Microorganisms. 2022 Apr 8;10(4):790. doi: 10.3390/microorganisms10040790 (PMC9030170; doi:10.3390/microorganisms10040790)

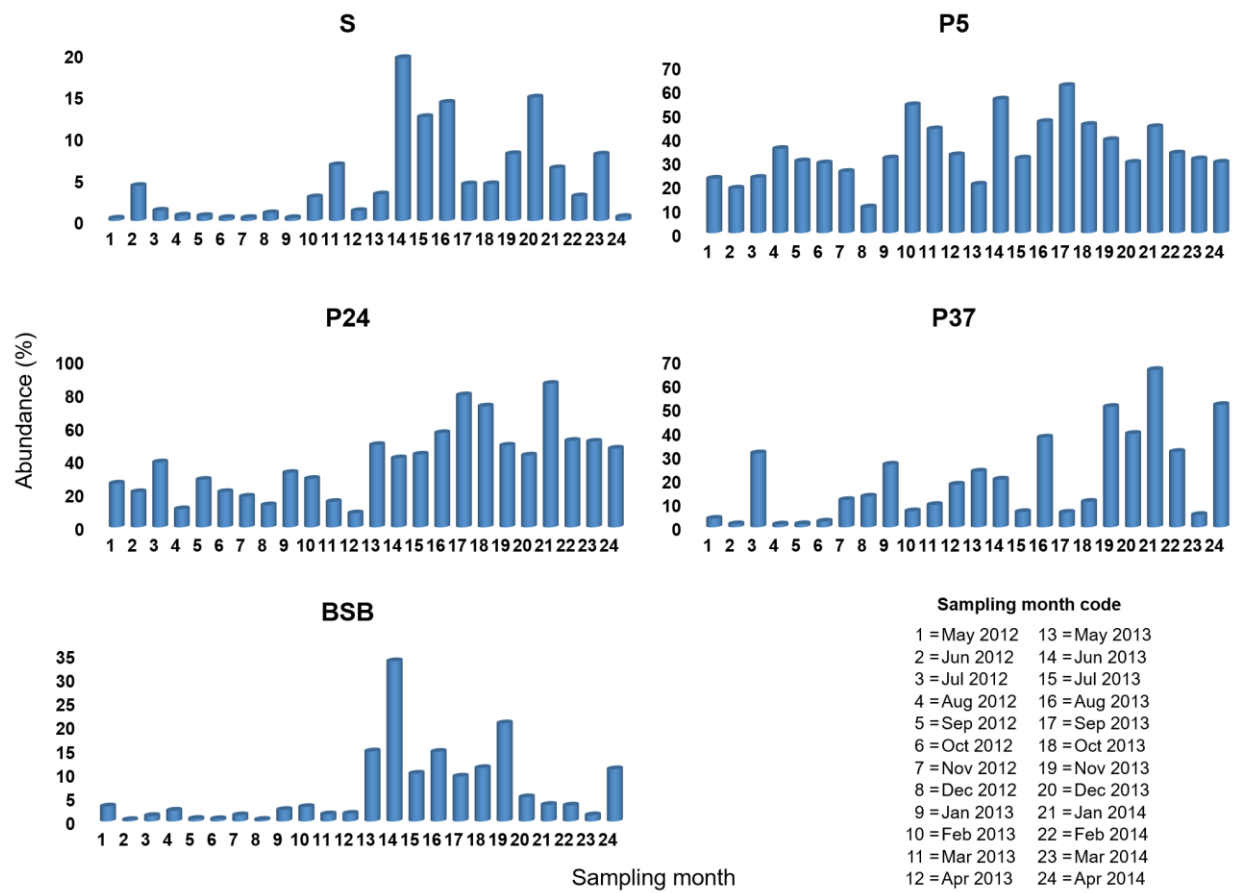

**Figure S1.** Occurrence of *Pontimonas* among the ST sampling sites in the period May 2012–April 2014.

Supplement: Supplementary file 1 [file microorganisms-10-00790-s001.zip › microorganisms-1631548 - Figure S1.pdf]
